# The Circadian Composition of Breast Milk: A Natural Starting Point for Chrononutrition

**DOI:** 10.1007/s13668-026-00749-1

**Published:** 2026-03-06

**Authors:** Sena Aksu, Sabriye Arslan

**Affiliations:** 1 Faculty of Health Sciences, Department of Nutrition and Dietetics, Toros University, Mersin, 33140 Türkiye; 2 Institute of Health Sciences, Department of Nutrition and Dietetics, Gazi University, Ankara, 06490 Türkiye; 3 Faculty of Health Sciences, Department of Nutrition and Dietetics, Gazi University, Ankara, 06490 Türkiye

**Keywords:** Chrono-nutrition, Breast milk, Breast milk composition, Circadian rhythm, Circadian feeding

## Abstract

**Purpose of Review:**

Breast milk is a highly bioavailable biological fluid that provides all essential fluids, macro and micronutrients, growth factors, and bioactive substances necessary for optimal growth and development of infants. Chrononutrition is a concept that examines the effects of the timing of food intake on physiological rhythms. This review aims to summarize current evidence on the circadian composition of breast milk and its potential implications for infant chrononutrition and early-life metabolic programming.

**Recent Findings:**

Fluctuations in the composition of breast milk across a 24-hour period, particularly the variations between night and day milk, and infants’ consumption of milk with this changing composition, contribute to breast milk being considered a form of chrononutrition. Thus, infants affected by signals from their mothers during the prenatal period continue to regulate their circadian rhythms by connecting with their mothers after birth. The circadian variation in breast milk is best characterized by higher concentrations of melatonin and tryptophan in night milk and elevated cortisol levels in day milk. Beyond these neuroendocrine factors, the concentrations of macro and micronutrients and immunological components have also been shown to exhibit diurnal fluctuations.

**Summary:**

These chrononutritional properties of breast milk may contribute to the regulation of infants’ sleep-wake cycles and support growth and development.

## Introduction

 Breast milk is a complex, highly bioavailable biological fluid that provides all the essential components required for the healthy growth and development of the infants [1]. Breast milk contains both nutritive and bioactive components, such as bacteriostatic factors, oligosaccharides, vitamins, minerals, amino acids, digestive enzymes, hormones, lipids, and growth factors [2]. For this reason, organizations such as the World Health Organization (WHO) and United Nations Children’s Fund (UNICEF) recommend that infants exclusive breastfeeding for the first six months of life, with continued breastfeeding alongside complementary until at least two years of age [3, 4].

The composition of breast milk is dynamic and influenced by several factors, including the lactation stage, the timing of infant feeding, and the circadian rhythms. The human body generally follows a 24-hour circadian rhythm directed by the suprachiasmatic nucleus located in the hypothalamus [1]. Chrononutrition is known as the circadian timing of nutrition and argues that meal timing, frequency, energy distribution, and regularity affect the body’s circadian rhythm. It is desirable to align the eating pattern with the body’s biological rhythm [5, 6]. In this regard, the concept of chrononutrition is based on a circadian system that supports wakefulness and eating during the biological day and sleep and fasting during the biological night [5].

Chrononutrition emphasizes the importance of consuming the most appropriate type and amount of food at the most appropriate time of day for maintaining optimal health [7, 8]. Since the relationship between chrononutrition and health indicators in adults has attracted attention, its effects on newborns should also be evaluated [9, 10]. In this regard, the newborn’s circadian rhythm can be determined by external factors such as exposure to light/darkness and feeding times, and may also be affected by differences in the composition of breast milk (especially bioactive components) throughout the day in accordance with the mother’s circadian rhythms [11]. As a result, fluctuations in breast milk composition may provide the infant with information about the time of day, thereby regulating various basic functions such as metabolism, sleep, and hormone release. For this reason, breast milk exhibits a circadian variation that may represent a powerful form of chrononutrition [7, 11].

In this context, our review will evaluate the current literature on infants’ circadian rhythms, chrononutrition, and breast milk. In addition to previous systematic reviews, recently published studies have also been taken into account, aiming to examine the circadian variation in breast milk and how it differs from other alternative feeding methods. The dates and contents of the most recent systematic reviews and overviews are specified, and additional literature published is presented in Table 1. In doing so, this review intends to provide a more up-to-date and comprehensive perspective to the existing literature.

**Table 1:** Recent systematic reviews and additional studies published thereafter on the circadian variation of human milk components

Year	Authors	Type	Focus
2019	Hahn-Holbrook et al.	Review	Discusses how circadian variations in human milk may help promote healthy infant circadian biology.
2020	Italianer et al.	Systematic Review	Full overview of human milk compounds exhibiting circadian variation.
2020	Moyo et al.	Review	Current knowledge on the circadian variation of breast milk nutrients and hormones.
2022	Caba-Flores et al.	Review	Highlights the importance of chrononutrition in relation to breast milk.
Following these reviews, 15 studies conducted after the systematic review in particular, which reported different findings regarding the circadian variation of certain components, are listed below.			
2007	Aparicio et al.	Longitudinal study	Examined the effects of chronologically separated milk with different tryptophan concentrations on the activity–rest cycles of 12–20-week-old infants adapted to light/dark conditions.
2007	Cubero et al.	Randomized Controlled Trial	Investigated the effect of day/night-separated formula on nighttime sleep.
2019	Qin et al.	Longitudinal study	Investigated factors associated with melatonin concentrations and circadian rhythms in breast milk of both preterm and term infants.
2020	Paulaviciene et al.	Cross-sectional study	Examined circadian variations in human milk macronutrients and energy content among mothers who delivered at different gestational ages.
2020	Toorop et al.	Longitudinal study	Explored the relationship between 24-hour exposure to milk glucocorticoids at 1 month postpartum and infant behavior and sleep at 3 months.
2021	Datta et al.	Longitudinal study	Assessed the impact of obesity, circadian rhythm, and prolonged breastfeeding on endocannabinoid levels in human milk.
2021	Mank et al.	Longitudinal study	Investigated the natural time course of human milk insulin concentration during the first ten postpartum days.
2021	Zietek et al.	Cross-sectional study	Identified corticosterone as a regular human milk component and evaluated the concentration correlation between sodium and potassium in breast milk.
2023	Suwaydi et al.	Longitudinal study	Provided new insights into circadian variation (24 h) and between-feeding differences in leptin, adiponectin, insulin, fat, and glucose concentrations in breast milk.
2024	Gogel et al.	Longitudinal study	Assessed effects of time of day, feeding duration, and breast-to-breast differences on total protein, lactose, individual proteins, and total lipids in human milk.
2024	Zielinska-Pukos et al.	Longitudinal study	Investigated energy value and macronutrient content of breast milk during the first six months of breastfeeding, providing updated information.
2024	Soledad et al.	Longitudinal study	Analyzed circadian behavior of specific immune cell populations and proinflammatory cytokines in transitional milk of preterm infants.
2024	Taufek et al.	Longitudinal study	Determined the iodine concentration of human milk.
2025	Taufek et al.	Longitudinal study	Investigated circadian patterns of zinc, copper, selenium, and bromine in human milk throughout six months postpartum and reported new findings.
2025	Woortman et al.	Longitudinal study	Investigated circadian variations of oxytocin, IgA, and lactoferrin in human milk

## Composition of Breast Milk and its Interaction with the Biological Clock

**Fig. 1:**
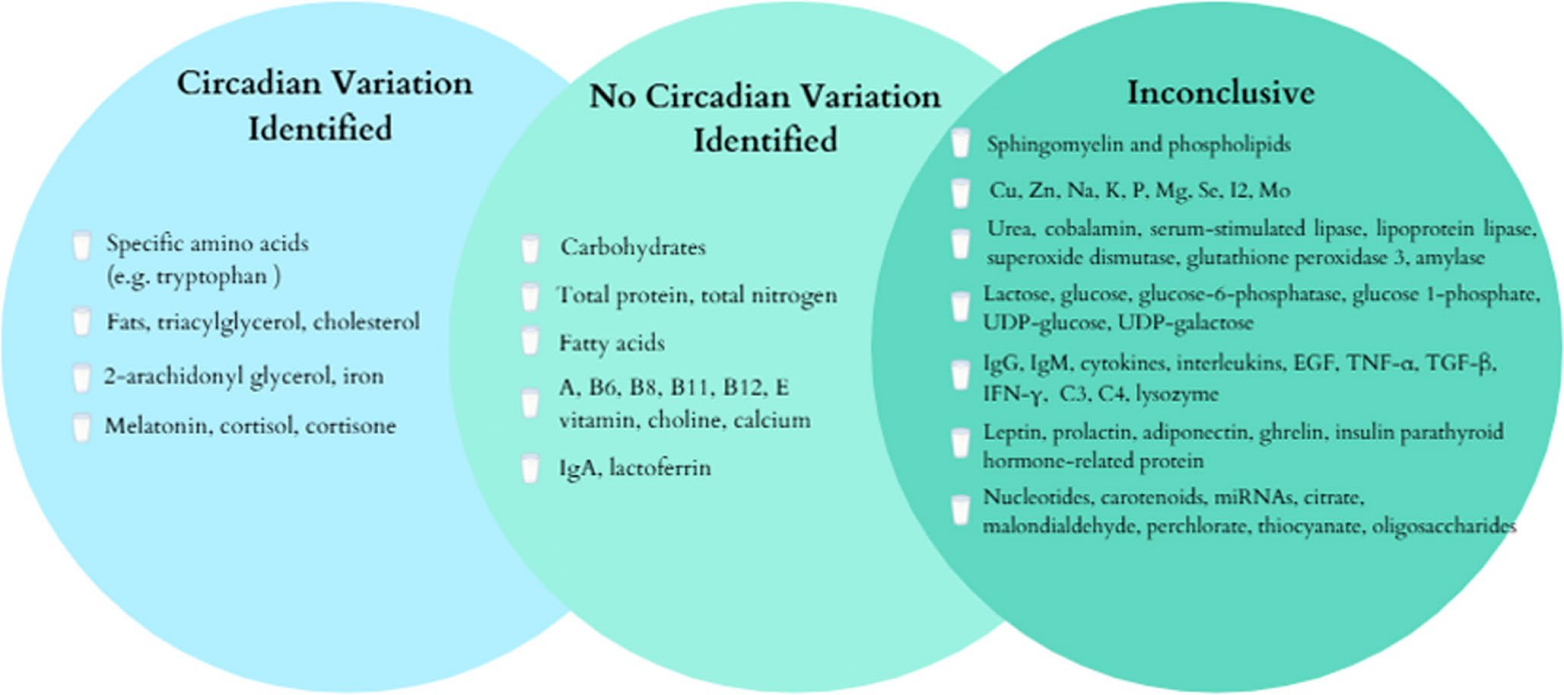
Circadian Variation in Breast Milk Components. The components of breast milk have been grouped according to whether or not they show circadian variation. Abbreviations: UDP, uridine diphosphate; Ig, immunoglobulin; TNF-α,: tumor necrosis factor alpha; TGF-β: Transforming growth factor beta; IFN-γ: interferon gamma; EGF, epidermal growth factor; C3 and C4, Complement components 3 and 4. (Information obtained from [1, 3, 17] sources has been modified and created at Canva.com.)

**Fig. 2:**
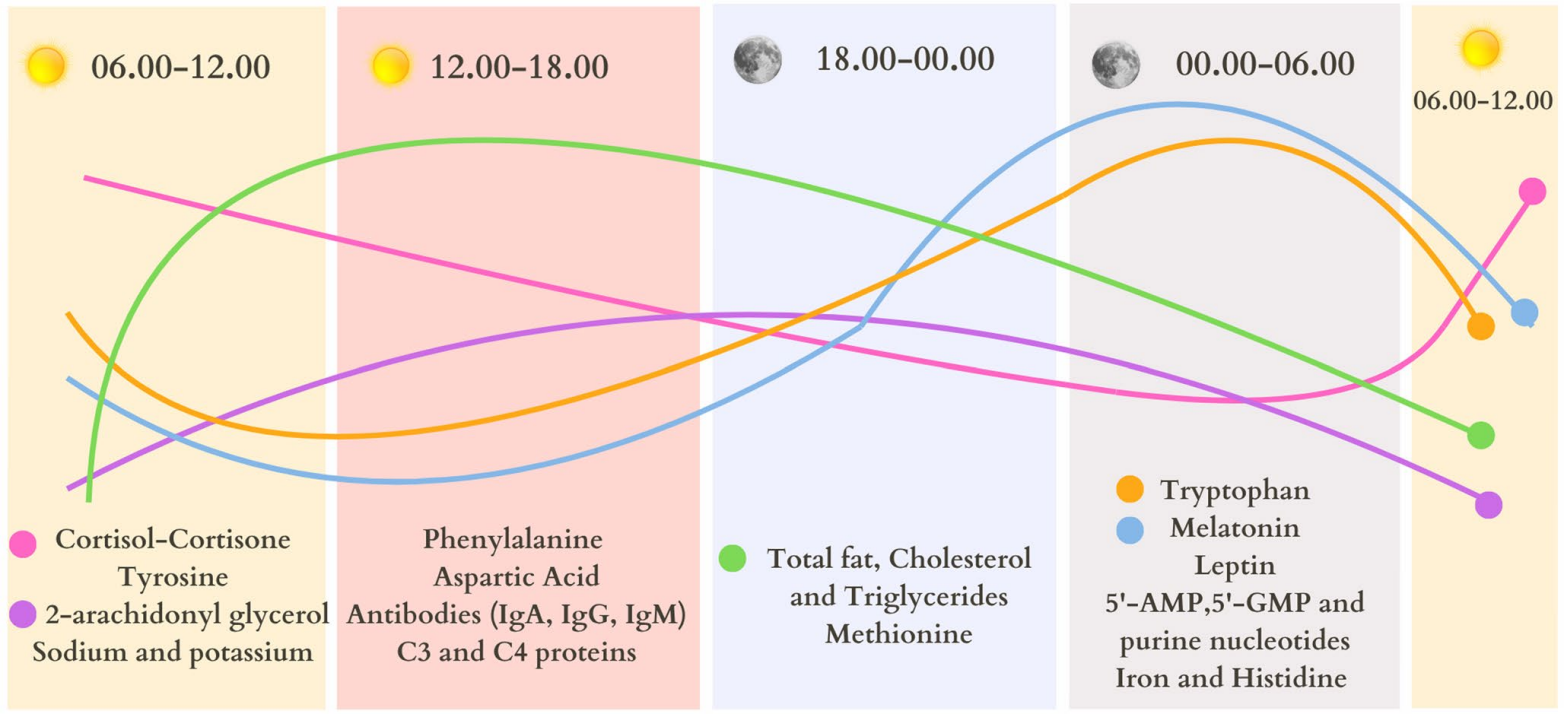
(Graphical Abstract) Circadian variation of breast milk components. Components reported to have a circadian rhythm in breast milk have been grouped according to time intervals. In addition, curves based on peak and trough values shown in the literature for these components over 24 h have been schematized. (Information obtained from [3, 7, 10, 17] sources has been modified and created at Canva.com.)

Mothers’ milk is widely recognized as the gold standard source of nutrition for newborns [4]. Its typical composition includes of 87% water, 7% lactose, 3.8% fat, and 1.0% protein, providing 65–70 kcal of energy per 100 mL, which is essential for the growth and development of infants [12, 13]. However, it is important to note that the composition of breast milk changes throughout the lactation period, with colostrum being produced in the first 5 days, followed by transitional milk (6–15 days), and finally mature milk (15 days until weaning) [4, 14]. Breast milk, which contains multifaceted biological compounds, including carrier systems that support the development of the immune and metabolic systems, contributes to the short- and long-term health of newborns [15]. Infants fed with breast milk generally show a lower likelihood of developing various acute and chronic conditions, such as middle ear infections, acute diarrhea, respiratory tract infections, sudden infant death syndrome, diabetes, obesity, asthma, and atopic dermatitis [13]. During the nine months in the womb, the fetus is exposed to the mother’s circadian, metabolic, physiological, and behavioral rhythms. However, at birth, this circadian environment is disrupted, and newborns are unable to develop their circadian rhythms immediately. Nevertheless, after birth, the baby receives rhythmic cues from the mother through fluctuations in breast milk composition, including nutritional, hormonal, and immunological factors factors [7, 16]. As a result, breastfed infants can develop their circadian rhythms to a large extent, following their mother’s own patterns, until they are approximately 2–3 months old [14]. At this point, Fig. 1 illustrates the circadian variations of macronutrients, micronutrients, and bioactive factors in breast milk, while Fig. 2 schematizes the fluctuations of these components according to time intervals.

### Nutritional Changes in Breast Milk Throughout the Day

Carbohydrates, one of the macronutrients found in breast milk, make up 60–70 g/L and account for 40% of the total energy reserve [13]. The main carbohydrate in breast milk is lactose, which can affect a baby’s appetite, feeding pattern, and body composition. Lactose is the most abundant macronutrient, with a concentration of 56 g/L in colostrum and an average of 68 g/L in mature milk [7]. Along with lactose, breast milk also contains oligosaccharides along with lactose and small amounts of monosaccharides such as glucose and galactose [18]. The lactose content, which increases slightly from colostrum to transitional milk and mature milk, maintains osmotic pressure with a constant concentration in mature milk [13]. These carbohydrates are broken down and absorbed by an enzyme called lactase [4]. Studies have shown that changes in breast milk carbohydrates do not help synchronize the newborn with its external environment. This is because breast milk carbohydrates undergo the least change, with relatively stable fluctuations over 24 h [1, 19]. Consistent with the 2020 meta-analysis, recent studies have also reported that carbohydrate concentrations remain relatively stable throughout the day, ranging from 6.7 to 6.9 g per 100 mL [3, 20]. In contrast, one study noted that, although no statistically significant differences were observed in preterm milk samples, more pronounced daily fluctuations occurred compared to term milk, with the highest concentration at night (7.40 g/100 mL) and the lowest during the day (7.32 g/100 mL) [19]. No circadian rhythm was observed in the total carbohydrate concentration of breast milk, and when lactose, glucose, and components were evaluated separately, circadian variation was rarely observed. It was noted that evidence for components such as glucose 6-phosphate, glucose 1-phosphate, UDP-glucose, and UDP-galactose was limited [3]. However, in a recent study, contrary to general knowledge, strong circadian rhythms were observed in glucose concentration, with the lowest levels occurring around 10:00 a.m [1]. The authors attributed this discrepancy to the small sample size and suggested that it may be partly due to a delayed response of maternal plasma glucose concentrations, which are stable between 1:00 a.m. and 5:30 a.m. in adults and begin to rise after 10:00 a.m., to these rhythms. Additionally, infants’ glucose intake began to decrease around 5:00 a.m. and increased after 1:00 p.m [1]. In this context, further studies on glucose could help prevent this confusion, and it could be determined whether a circadian rhythm exists.

Another macronutrient found in breast milk is protein, which is primarily made up of casein and whey proteins [12]. The ratio of whey to casein in breast milk changes throughout lactation, ranging between approximately 70:30 and 80:20 in early lactation, around 60:40 in mature milk, and balancing out at 50:50 in late lactation [4, 21]. These proteins in breast milk are essential for infants’ growth and development, as well as providing important bioactive proteins and peptides [13]. While protein makes up only about 1% of breast milk, it is highly concentrated (1.4–1.6 g/100 mL) in the first few weeks and gradually decreases (to 0.8–1.0 g/100 mL at three to four months and 0.7–0.8 g/100 mL after six months) [4, 12].

In contrast to carbohydrates, findings regarding circadian fluctuations in the protein content of breast milk are more inconsistent [19]. Recent meta-analysis indicates that the majority of studies (9 out of 11) report no significant circadian variation in total protein content while the few studies that do report variation may reflect false positive findings due to acrophase mismatch [3]. With the addition of studies to the literature in recent years, evidence supporting the absence of circadian fluctuations in total protein content is steadily increasing; breastfeeding in the first (1.3 g/100 mL and 1 g/100 mL, respectively), third (1 g/100 mL and 0.8 g/100 mL), and sixth (0.9 g/100 mL and 0.7 g/100 mL) months, while preterm milk samples tend to show slightly more pronounced circadian variations compared to term milk samples, this difference is likely attributable to sample size [19, 20]. However, some reports indicate that protein and proteome abundance tends to be higher in the evening and night, whereas others find the highest protein levels during daytime hours (1.37–1.43 g/100 mL at 12 PM and 6 PM). These inconsistencies may be influenced by various factors, including maternal dietary habits [19, 22].

However, it should be noted that among the amino acids present in breast milk, circadian fluctuations are observed particularly in tryptophan, with levels rising at night, peaking in the early morning hours, and reaching their lowest levels in the afternoon [7, 23]. This is because tryptophan is an amino acid that is a precursor to both serotonin and melatonin, and is a key amino acid involved in sleep/wake circadian rhythms [24]. Additionally, some individual amino acids may also show circadian variation in breast milk. As an illustration, it has been noted that neuroactive amino acids (tyrosine, methionine, phenylalanine, histidine, aspartic acid, and glycine) are at their highest levels in day milk [10, 16]. Nevertheless, this circadian variation appears to be more pronounced in mature milk compared to colostrum and transitional milk. For example, only methionine and tryptophan show daily variation in transitional milk, with the circadian rhythm occurring at approximately 3:00 a.m. for tryptophan and 6:00 p.m. for methionine [3, 10, 25]. The average acrophase times for other amino acids were determined as 12:08–12:33 for phenylalanine and aspartic acid, 09:38 for tyrosine, and 03:14 for histidine [25].

Another nutrient in breast milk is fat, which makes up nearly 50% of the baby’s nutritional intake. It is the second most common macromolecule in breast milk and plays a crucial role in the baby’s growth and central nervous system development [4, 13]. On average, fat makes up 3.5–4.5% of breast milk, with 95–98% of it in the form of triglycerides. However, almost half of the fatty acids in milk are saturated fatty acids [4, 13].

Although the average fat content of breast milk remains relatively constant during the first months of lactation, it can be by the mother’s diet, differences in eating habits, and breast fullness, including feeding time. Fat is the most variable macronutrient in milk [13, 26]. It increases with time or maturation. For example, the fat content of colostrum is 2.2 g/100 mL, which increases to 3.0 g/100 mL in transitional milk and 4 g/100 mL in mature milk [27]. Additionally, there is 2–3 times more milk fat in hind milk (end of feeding) compared to fore milk (beginning of feeding) after each breastfeeding session [26, 27]. One study reported that this difference averaged 24 g/L [21].

Breast milk exhibits circadian variation in fat content. According to a meta-analysis conducted in 2020 reported circadian variation in 15 of 19 studies on total fat concentration (with peaks in the evening hours), while the remaining studies yielded different results [3]. These differences may be explained by factors such as ethnic differences, maternal nutritional status, milk composition measurement techniques, and population characteristics [28]. Recent studies also show heterogeneity: some report no distinct circadian rhythm in total fat during the early stages of mature milk, whereas others indicate that fat concentration increases between 10:00 a.m. and 8:00 p.m. (peaking around 5:00 p.m.) and decreases between 8:00 p.m. and 8:00 a.m [1, 19, 29].

The circadian variation in fat concentration may be influenced by changes in milk volume in the udder before and after each feeding [1]. However, few studies have examined circadian patterns in fore milk and hind milk, with the only study assessing both reporting that total fat increased throughout feeding and was higher at midday compared to nighttime [3, 22]. Similarly, in both preterm and term infants, night milk has been shown to contain higher total fat than day milk, with peak concentrations observed at 12:00 p.m. and 6:00 p.m [10, 19]. Findings regarding the lactation stage are inconsistent; approximately half of the studies report circadian changes (with one study noting no change at six months), while the other half report no such effect [20, 30–32]. This inconsistency may stem from differences in sample collection periods (ranging from 3 to 30 days to 1–6 months), different populations (Thailand, Brazil, Switzerland, and Poland), and technical variations in the methods used (such as Creamatocrit and Human Milk Analyzer).

When triacylglycerols, which constitute the majority of fat, were examined separately in a meta-analysis (in two of three studies), it was revealed that levels were low in the morning, peaked in the afternoon or evening, and exhibited consistency with total fat content [3]. In a similar manner, the investigation of circadian variation in relation to cholesterol revealed a peak in the evening in three out of four studies [3]. Nonetheless, although previous studies have reported daily changes in total TAG levels and cholesterol in milk, there is less information about the daily regulation of lipids found in low amounts, such as sphingolipids, glycerolipids, and phospholipids [3, 33]. In this context, although while studies conducted in meta-analyses suggest that a consistent circadian rhythm is not observed, possibly due to small sample sizes, a recent study reported that sphingolipids and phospholipids exhibit higher concentrations in the evenings, with diacylglycerols and triacylglycerols showing more pronounced circadian variation compared to phospholipids and sphingolipids [3, 33]. It is hypothesised that this elevated concentration is associated with the augmented total fat content of breast milk during the evening hours [17]. In summary, it can be posited that the levels of fat in breast milk exhibit a diurnal pattern, with a surge in the morning, a peak between midday and evening, and a decline at night, following circadian rhythms [7].

Breast milk contains sufficient amounts of vitamins and minerals to support normal infant growth during the first six months, with a few exceptions (such as vitamins D and K) [4, 34]. However, a comparison of milk from different stages of lactation reveals that colostrum is rich in whey proteins and minerals but contains lower levels of lactose, fat, and certain vitamins compared to mature milk [12]. In comparison with mature milk, the levels of chloride, sodium, and magnesium are higher, whilst calcium and potassium are lower [12].

Though there is a lack of evidence regarding the circadian rhythms of vitamins, the literature indicates that no circadian variation has been observed in vitamins A, B1, B2, B3, B6, B8, B12, E, folate, and choline, and no recent studies have reported otherwise [3]. Minerals in breast milk, particularly iron concentrations (which fluctuate continuously throughout the evening or night with acrophase), show strong evidence of circadian variation, while evidence for other trace elements (calcium, copper, sodium, zinc, potassium) remains inconsistent [3]. Regarding other minerals, the literature indicates that zinc, sodium, and potassium may exhibit circadian rhythms; evidence for phosphorus, magnesium, iodine, and molybdenum remains conflicting or limited; and calcium and copper generally do not display circadian variation [3, 10]. In this regard, recent studies showing that circadian changes in sodium and potassium (at their highest levels in the morning) change in tandem with the cortisol concentration in breast milk (where rising cortisol corresponds to decreasing sodium and increasing potassium), while reporting that copper and iodine exhibit similar circadian variation throughout the day in a “V” pattern [35–37]. However, it has been reported that zinc and bromine also exhibit circadian variation in a “V” pattern, whereas selenium exhibits inconsistent circadian changes during the first six months [36]. These differing findings may be attributed to variations in lactation stages across studies, the extension of analyses over longer periods, increased sample numbers and sampling frequency, population- and mother-specific dietary differences (particularly mineral supplementation), and inconsistencies in sample collection protocols.

### Immunological Changes Occurring in Breast Milk Throughout the Day

In addition to nutritional biomolecules, breast milk contains a plethora of bioactive components that facilitate the survival and health of the infant [13]. The provision of various bioactive factors, including but not limited to lactoferrin, lysozyme, leukocytes, immunoglobulins, cytokines, hormones, stem cells, breast milk oligosaccharides, microRNAs, antioxidants and growth factors plays a vital role in strengthening the baby’s immune system [11, 12]. Nonetheless it has been demonstrated that colostrum exhibits higher levels of immunological components in comparison to mature milk [12, 13].

Although some circadian changes have been reported for bioactive components, the current evidence is still limited for drawing definitive conclusions [17]. Accordingly, findings on immunological components such as immunoglobulins (IgA, IgG, and IgM), interferons, lactoferrin, transforming growth factor-β (TGF-β), and tumor necrosis factor-alpha (TNF-α) have been reported inconsistently [3]. In this context, recent studies have shown that in colostrum and mature milk, IL-2, IL-10, and IFN-γ are higher at night, while IL-6 and TNF-α are higher during the day, exhibiting circadian rhythms; in preterm transitional milk, immune cell populations and proinflammatory cytokines display more pronounced rhythmic fluctuations during the day; in contrast, components such as oxytocin, IgA, and lactoferrin do not show diurnal variation [38–40]. However, the current evidence remains limited, and definitive conclusions in this area have not yet been reached.

In addition, the total nucleotide rhythms in breast milk (generally highest in the evening and lowest in the morning) are also noteworthy. However, individual nucleotides known to be important for GABA and melatonin release [adenosine 5’monophosphate (5’AMP), guanosine 5’-monophosphate (5’GMP), uridine 5’-monophosphate (5’UMP), cytidine 5’-monophosphate (5’CMP), and inosine 5’-monophosphate (5’IMP)] [41]. It is evident that 5’AMP and 5’GMP manifest substantial circadian rhythms during the dark period (at 20:19 and 05:08, respectively), while 5’CMP and 5’IMP demonstrate notable circadian rhythms during the day (at 18:40 and 19:14, respectively). Despite the absence of a discernible circadian rhythm in 5′UMP levels, a nocturnal increase has been observed [41]. Among other bioactive factors, no definitive conclusions have been reached regarding the presence of a circadian rhythm related to microRNAs, breast milk oligosaccharides, citrate, and malondialdehyde in breast milk [3].

### Hormonal Changes Occurring in Breast Milk Throughout the Day

Hormones such as leptin, adiponectin, ghrelin, insulin, melatonin, and glucocorticoids in breast milk play a significant role in regulating infant growth and body composition development [42]. For this reason, the circadian changes in the endocrine components of breast milk are noteworthy [17]. Notably, hormone concentrations in breast milk exhibit greater susceptibility to rhythmic variations compared to other components, with each hormone following a distinct pattern of change [17].

Of these, melatonin is a neurohormone with immunomodulatory and antioxidant functions, as well as a role in circadian rhythm regulation linked to sleep patterns [43, 44]. The establishment of the day-night rhythm for melatonin secretion from the pineal gland typically takes two to three months post-birth, which may result in newborns lacking sufficient melatonin during the early postnatal period. In this context, newborns rely on exogenous melatonin, at least from breast milk [45].

Melatonin, which is closely linked to immune system, exhibits a progressive decrease throughout lactation in both vaginal and cesarean-section deliveries, with levels being notably higher in colostrum [7, 44]. Additionally, melatonin levels during pregnancy have been shown to influence fetal development [46]. Several factors, including maternal health, environmental conditions, gestational age, and feeding practices, can influence melatonin concentrations in breast milk [45].

The circadian rhythmicity of melatonin in breast milk is one of the most extensively researched topics in this area. The general consensus in the literature is that melatonin levels are high throughout the night (peaking between midnight and 3 a.m.) and negligible during the day (below the detection limit between 2 p.m. and 5 p.m.), with the circadian rhythm maintained even during different stages of lactation [7, 17, 47]. Considering both meta-analysis and review findings, melatonin levels in colostrum are reported to be 15.5 pg/mL during the day and 42.75 pg/mL at night; in mature milk during the first six months, they are 12.56 pg/mL during the day and 39.8 pg/mL at night, with levels averaging 46.9 ± 4.2 pg/mL throughout the night [3, 48]. Furthermore, the fact that melatonin’s acrophase occurs several hours after the acrophase of tryptophan, its metabolic precursor, highlights the existence of a timing system [3].

Glucocorticoids, along with melatonin, help signal the body’s internal clock by indicating the time of day and night, correlating with wakefulness and sleep phases [7]. The timing of the establishment of these circadian rhythms varies considerably during the first 3–6 months of life, a period when circadian alignment remains fragile and prone to disruptions [45]. In addition, glucocorticoids in breast milk (cortisol and its inactive form cortisone) are closely linked to signaling pathways for processes such as energy balance, regulation of stress activity and immune function in the infant [17]. However, cortisone is much higher in breast milk than cortisol due to increased expression in the mammary glands [29].

Both cortisol and cortisone levels follow a daily rhythm, peaking in the morning (daytime) and corresponding with the maternal hypothalamic-pituitary-adrenal axis activity [49]. In one of the studies, it was emphasized that cortisol and cortisone concentrations were higher in the morning between 04.00 and 10.00 (2.97 ng/ml and 4.88 ng/ml), in the afternoon (1.20 ng/ml and 3.54 ng/ml), in the evening (0.69 ng/ml and 2.13 ng/ml) and at night (1.59 and 3.27 ng/ml) [50].

The earliest reports of circadian patterns for cortisol in infants suggest they can begin to emerge as early as two months, though this can extend to nine months [10]. Beyond this information, some studies have observed no association between the rhythmicity of breast milk glucocorticodies at 1 month and infant behavior, sleep, growth and body composition at 3 months [51, 52]. These studies proposes that this discrepancy may be attributed to the lack of serial cortisol measurements and incomplete data on body composition [51, 52]. It was also thought that the effect of the circadian rhythm of breast milk glucocorticoids on infants could be explained by macronutrient composition, but no relationship was found [29].

Circadian rhythms are also observed in endocrine components found in breast milk such as leptin, prolactin, ghrelin, adiponectin, and insulin, although there is limited evidence [3]. Among these hormones, leptin is the most widely studied appetite hormone apart from melatonin and cortisol [53]. Leptin, which is thought to help regulate metabolic programming by balancing infants’ energy intake and appetite, was observed to have significantly higher concentrations between 10:01 p.m. and 4:00 a.m. in a study conducted using fat-free breast milk, supporting the presence of nocturnal increases [54, 55]. However, because leptin levels are generally higher in whole milk, extending the analysis to whole breast milk samples was considered. A recent study reported that leptin levels peaked around 05:00, decreased between 12:00 and 17:00, and then increased again between 20:00 and 06:00 [1, 55].

Prolactin, another hormone found in breast milk, is thought to help nutrient absorption in the intestines of infants [10]. One study shows that there is a temporal variation in the concentration of the hormone prolactin, with the concentration being highest in the early morning (between 02.01 and 06.00 h). This concentration was then shown to be lowest between 10.01 and 18.00 h [56]. Circadian variation in breast milk ghrelin, which is thought to play a role in infant growth rate, energy homeostasis, weight gain and early programming, has not yet been studied in humans. However, rodent studies may offer insights into potential changes in breast milk [42]. In this context, in a study on 30 lactating rat mothers, ghrelin concentration in milk showed a significant rhythmicity only on day 5, peaking at 16:00 and 4:00 with a bimodal pattern. In the same study, the levels of leptin (on day 5) and adiponectin (on days 5 and 10) in milk also showed a similar rhythmicity (a biomodal pattern) over a half-day period [57]. Further longitudinal studies are still needed to clarify the potential functional implications of the circadian rhythms of these hormones on infant development.

Adiponectin is present in breast milk in higher concentrations than many hormones, including adipokines such as ghrelin and leptin [58]. Insulin has regulatory functions in glucose metabolism. In this context, adiponectin, leptin and insulin in breast milk may contribute to the protective effect against childhood obesity [59]. However, higher leptin concentrations and intermediate insulin concentrations are associated with lower weight-for-height in the first year of life [59]. Although research on the circadian rhythms of adiponectin and insulin levels in breast milk is limited, a recent study reported that both hormones exhibit strong circadian patterns, increasing between 10:00 and 20:00 and decreasing between 22:00 and 07:00 [1]. Another study evaluating insulin concentrations only during the first ten days postpartum similarly found that the rhythm was characterized by a decline during the night and an increase in the morning [60]. In addition, in the study assessing hormone intake, infant intake was calculated by multiplying the average concentration of breast milk samples collected before and after feeding by the corresponding feeding volume. The results showed that leptin and insulin intake (increasing from approximately 1:00 PM and decreasing after midnight) exhibited significant circadian rhythms consistent with their 24-hour concentration patterns. In contrast, the circadian rhythm of adiponectin intake (a clear decrease after ~ 05:00 and an increase after 15:00) was reported to be the opposite of the circadian rhythm of its concentration. The authors suggest that these findings may reflect the influence of circadian rhythms on breast milk volume and the associated changes in fat concentration [1].

Among the various neurochemicals found in breast milk, 2-arachidonoyl glycerol (2-AG) plays a role in the regulation of various physiological and cognitive processes of the infant [7, 17]. A recent study observed that 2-AG levels in milk exhibited a diurnal rhythm in both normal and obese mothers, with higher levels found in day milk (06:00–22:00) compared to night samples (23:00–5:00) [61]. The elevated levels of 2-AG reflect maternal plasma levels and may influence infant food intake and body mass index [7]. Overall, it is suggested that the hormones and immune factors transmitted through breast milk aid infants in developing their own circadian rhythms during the early months of life [61].

## Chrononutrition of Breast Milk and its Effects on Infant Health

Chrononutrition is the adjustment of nutritional quality and intake to coordinate with the individual’s biological clock [7]. Breastfeeding as a form of chrononutrition may offer short- and long-term health benefits on infant health, including sleep, metabolism and neurocognitive development, with breastfeeding aligned with circadian rhythms. Fetal circadian rhythms begin to develop around 30 weeks of pregnancy [10, 62]. However, infants are not born with a fully developed circadian clock, aside from daily fluctuations in body temperature. Rhythms such as rest-activity, sleep-wake and hormonal cycles develop slowly in the first months of life [10, 62]. Breastfeeding infants at these times can allow them to receive timed cues from their mothers, allowing the circadian rhythm to develop and mature [62]. In this regard, feeding models based on a chronobiological approach using breast milk are thought to potentially support the development of circadian rhythms in both preterm and term newborns [63, 64].

Breast milk is a dynamic fluid that adapts to the nutritional and immunological needs of the baby not only during breastfeeding but also during the day [65, 66]. However, when breast milk is unavailable, formula milk can become a staple food for the newborn [12]. For this reason, the design of the milks is as similar to breast milk as possible [65]. However, the circadian fluctuations in bioactive compounds that convey chronobiological information from mother to child may not be present in formula-fed infants [67]. The impact of the daily rhythms on infants have not been fully clarified, and ideas are being generated based on ongoing studies. For example, an infant fed with breast milk may exhibit more favorable physiological development, a better sleep/wake rhythm and therefore a more optimal sleep pattern compared to an infant fed with formula or by bottle [29, 67]. However, within this context, a systematic review and a meta-analysis have reported that exclusively breastfed infants wake more frequently at night [68, 69], while there is no difference in night or 24-hour sleep duration compared to formula-fed infants [68]. It is known that breastfed infants feed more frequently and that breastfeeding sessions are longer; this may affect both the infant’s and the mother’s nighttime awakenings [69, 70], and due to frequent awakenings, breastfed infants are thought to be exposed to time cues more often, potentially serving as a strong zeitgeber.

It was observed that breastfed infants began to develop their circadian rhythms in the second and third weeks, with clear patterns emerging by the sixth week, whereas mixed-fed infants were able to establish circadian rest-activity rhythms only by 12 weeks [46]. Overall, the rhythmic changes of melatonin, glucocorticoids, and specific amino acids and nucleotides in breast milk (involved in regulating infants’ sleep/wake cycle), as well as 2-AG and lipid components (supporting neurodevelopment and cognitive performance), play an important role in infant development [17]. Another important finding in this context, as demonstrated in a meta-analysis, is the difference in weight gain between breastfed and formula-fed infants, with higher weight gains observed in those fed by bottle or formula [71].

Breastfed infants may have stronger time cues, such as different components in formula milk, differences in maternal circadian rhythm, maternal contact activity, and feeding time, which may be the reason for the earlier development of circadian rhythm [46]. For instance, in one study, observed that melatonin in breast milk was absent in formula, suggesting it may help improve sleep and reduce colic compared to formula-fed infants [72]. Similarly, blunted cortisol rhythms in infants with colic indicate a possible disruption or delay in circadian rhythm development [73].

When breast milk is not available, donor breast milk is another suitable food. Milk delivered to milk banks is pasteurized (Holder pasteurization at 62.5 °C for 30 min) to neutralize any microbial agents [74]. In this regard, studies on the effect of pasteurization on melatonin levels in breast milk have reported conflicting results, with one study of 18 samples finding no change [75], while another study of 10 samples observed a significant decrease [76]. When direct breastfeeding is not possible, expressed breast milk can be administered according to circadian rhythms, either in 12-hour night and day cycles or divided into four six-hour phases: night, day, dusk, and dawn [63, 64]. This model is suggested to be associated with shorter hospital stays and significant improvements in anthropometric and physiological parameters [63].

Both in donor breast milk and in pumped milk, feeding times that are out of sync may negatively affect the development of the circadian time and sleep homeostasis [3, 77]. For example, if the mother gives her baby milk in the evening that she previously expressed during the day, the baby will not receive nocturnal time cues such as melatonin [77]. In this context, a preliminary study showed that breast milk expressed at the wrong time was significantly associated with a delay in infants’ time to fall asleep, supporting the idea of a potential effect on circadian rhythm [65]. At this stage, it is thought that day/night formulas designed according to the principles of chrononutrition may help reinforce infants’ sleep/wake rhythms, this view is supported by studies in which differentiated formulas with day- and night-specific nutrient compositions (lower tryptophan and higher protein with cytosine-5P, guanosine-5P, and inosine-5P during the day, and higher tryptophan and carbohydrates with adenosine-5P and uridine-5P at night) as well as tryptophan-enriched (3.4% tryptophan) night milk were associated with improvements in infants’ sleep parameters [67, 78].

## Conclusion and Recommendations

After birth, newborns do not have fully developed circadian rhythms due to the immaturity of their neural control mechanisms [57]. Consequently, they benefit from breast milk, a potent form of chrononutrition, with its composition varying according to the mother’s circadian rhythms. Thus, infants, who are influenced by maternal signals in the prenatal period, acquire their rhythms in the postnatal period by connecting with the mother through breast milk as well as environmental signals [62, 77]. Components in breast milk that regulate the infant’s circadian system may play a role by containing higher concentrations during the day or night [49]. Key components include melatonin, glucocorticoids, tryptophan, lipids, triacylglycerol, cholesterol, and iron [3]. Furthermore, knowledge of circadian variations in the concentrations and intakes of all macro-micro components and bioactive substances in breast milk will enable further investigation of their impact on infant health and development [1]. Disruptions in circadian rhythms can affect the sleep-wake cycle of infants and may lead to long-term health consequences, including impaired glucose tolerance, cardiometabolic diseases, and psychiatric disorders [79]. In addition to the systematic reviews in the literature (particularly the study by Italiner and colleagues), recent research has revealed promising findings regarding the circadian rhythms of components such as glucose, adiponectin, leptin, prolactin, zinc, sodium, 2-AG, potassium, copper, and iodine. In contrast, results related to total protein, total fat, ghrelin, and cytokines remain inconsistent, while evidence suggesting the absence of a circadian rhythm for IgA and lactoferrin has increased. It has been observed that recent studies, by employing different methodological approaches, such as calculating infants’ intake of specific components and conducting long-term evaluations extending to the sixth stage of lactation, have brought new perspectives to the field and yielded novel findings. High-quality studies are needed to examine many understudied breast milk components (especially those whose circadian variation cannot be determined with certainty) to enrich knowledge on breast milk chronobiology. Future research focused on these circadian variations in breast milk composition could lead to more comprehensive insights into infant health and aid in the optimal development of infants’ circadian rhythms. In this context, it is emphasized that the Chronobiological Feeding Model should be addressed more extensively in studies and that its effects on infants should be examined in a more comprehensive manner. Additionally, based on these studies, alternative food sources -such as formula milks with day- and night-appropriate ingredients and knowledge of donor milk timing- could be developed to help provide circadian rhythm support for infants who cannot be breastfed.

##  Key References

- Suwaydi, M.A., et al., *Circadian variation in human milk hormones and macronutrients.* Nutrients, 2023. **15**(17): p. 3729. https://doi.org/10.3390/nu15173729.
  - ◦This article covers a significant study that contributes to the understanding of circadian variations in hormones and macronutrients in breast milk. Moreover, it provides contributions that may support and potentially prompt a reconsideration of findings from recent meta-analyses on the subject.
- Cui, M., et al., *The chrononutrition of human milk: role of the circadian variation in human milk composition on sleep-wake regulation and brain development of infant.* Current Opinion in Food Science, 2025: p. 101283. https://doi.org/10.1016/j.cofs.2025.101283.
  - ◦In this article, the circadian variations in human milk components are discussed, and their potential physiological significance, particularly in relation to infant neurodevelopment, is evaluated.
- Caba-Flores, M.D., et al., *Breast milk and the importance of chrononutrition.* Frontiers in Nutrition, 2022. **9**: p. 867507. https://doi.org/10.3389/fnut.2022.867507.
  - ◦ This article examines the diurnal variations of nutritive and non-nutritive components in human milk; it sheds light on the physiological significance of these variations in infants as well as the effects of the environmental light/dark cycle on neonates.

## Data Availability

No datasets were generated or analysed during the current study.
